# Seroprevalence of *Borrelia burgdorferi* sensu lato and *Anaplasma phagocytophilum* Infections in German Horses

**DOI:** 10.3390/ani13121984

**Published:** 2023-06-14

**Authors:** Heidrun Gehlen, Katharina Inerle, Alexander Bartel, Sabita Diana Stöckle, Sebastian Ulrich, Beatrice Briese, Reinhard K. Straubinger

**Affiliations:** 1 Equine Clinic: Surgery and Radiology, Freie Universitaet Berlin, 14163 Berlin, Germanysabita_diana.stoeckle@vetmed.uni-leipzig.de (S.D.S.); beatrice.briese@synlab.com (B.B.); 2 Institute for Veterinary Epidemiology and Biostatistics, Freie Universitaet Berlin, 14163 Berlin, Germany; alexander.bartel@fu-berlin.de; 3 Chair of Bacteriology and Mycology, Department of Veterinary Sciences, Faculty of Veterinary Medicine, Institute for Infectious Diseases and Zoonosis, Ludwig-Maximilians-Universität, 85764 Oberschleißheim, Germany; s.ulrich@lmu.de (S.U.); r.straubinger@lmu.de (R.K.S.)

**Keywords:** equine Lyme borreliosis, equine granulocytic anaplasmosis, seroprevalence, co-infection

## Abstract

**Simple Summary:**

There are limited data on Lyme borreliosis, a tick-borne disease caused by the bacteria of the *Borrelia burgdorferi* sensu lato complex, in horses. Seropositivity is not necessarily associated with clinical disease. Data on seropositivity against *Borrelia burgdorferi* sensu lato and *Anaplasma phagocytophilum* in German horses are sparse. Therefore, serum samples from horses (*n* = 123) suspected of having Lyme borreliosis and clinically healthy horses (*n* = 113) residing at the same stables were tested for specific antibodies against *Borrelia burgdorferi* sensu lato and *Anaplasma phagocytophilum*. First, the horses were screened for antibodies against *Borrelia burgdorferi* sensu lato. Afterward, the horses were screened for antibodies against *Borrelia burgdorferi* sensu lato and *Anaplasma phagocytophilum* (SNAP^®^ 4Dx Plus^®^ ELISA). The clinical signs of suspect horses included lameness (*n* = 36), poor performance (*n* = 19), and apathy (*n* = 12). Twenty-three percent (*n* = 26) of suspect horses and 17% (*n* = 18) of clinically healthy horses were seropositive for having a *Borrelia burgdorferi* sensu lato infection (*p* = 0.371), showing that the detection of specific antibodies against *Borrelia burgdorferi* alone is not sufficient for a diagnosis of equine LB. *Anaplasma phagocytophilum*-seropositivity and seropositivity against both pathogens was 20%/6% in suspect horses and 16%/2% in the clinically healthy population, showing only minor differences (*p* = 0.108). Unspecific testing for antibodies against *Borrelia burgdorferi* sensu lato without clinical suspicion of Lyme borreliosis is not recommended since the clinical relevance of seropositivity against *Borrelia burgdorferi* sensu lato remains unknown.

**Abstract:**

There are limited data on Lyme borreliosis (LB), a tick-borne disease caused by the *Borrelia burgdorferi* sensu lato complex, in horses. Seropositivity is not necessarily associated with clinical disease. Data on seropositivity against *Borrelia burgdorferi* and *Anaplasma phagocytophilum* in German horses are sparse. Therefore, serum samples from horses (*n* = 123) suspected of having Lyme borreliosis and clinically healthy horses (*n* = 113) from the same stables were tested for specific antibodies against *Borrelia burgdorferi* sensu lato and *Anaplasma phagocytophilum*. The samples were screened for antibodies against *Borrelia burgdorferi* (ELISA and an IgG line immunoblot assay). Furthermore, the samples were examined for antibodies against *B. burgdorferi* and *Anaplasma phagocytophilum* with a validated rapid in-house test (SNAP^®^ 4Dx Plus^®^ ELISA). The clinical signs of suspect horses included lameness (*n* = 36), poor performance (*n* = 19), and apathy (*n* = 12). Twenty-three percent (*n* = 26) of suspect horses and 17% (*n* = 18) of clinically healthy horses were seropositive for having a *Borrelia burgdorferi* sensu lato infection (*p* = 0.371), showing that the detection of specific antibodies against *B. burgdorferi* alone is not sufficient for a diagnosis of equine LB. *Anaplasma phagocytophilum* seropositivity and seropositivity against both pathogens was 20%/6% in suspect horses and 16%/2% in the clinically healthy population, showing only minor differences (*p* = 0.108). Unspecific testing for antibodies against *B. burgdorferi* without clinical suspicion of Lyme borreliosis is not recommended since the clinical relevance of seropositivity against *Borrelia burgdorferi* sensu lato remains to be elucidated.

## 1. Introduction

The first cases of equine Lyme borreliosis (LB) were published in the USA in the state of Wisconsin [1,2], a region where *Borrelia burgdorferi* is endemic. Other cases were described in Europe shortly thereafter [3,4]. LB is a tick-borne (*Ixodes* spp.) disease that is difficult to diagnose in horses because the specific antibody response against organisms of the *Borrelia burgdorferi* sensu lato complex is not necessarily associated with clinical disease [5]. Studies on equine LB show varying seroprevalences in different geographical regions, depending on the distribution of infected *Ixodes* spp. ticks. However, it remains unclear whether a high antibody level in infected hosts correlates with clinical signs [6,7,8,9,10,11,12,13,14,15,16] or if there is no association between clinical signs and a positive titer [17,18,19,20,21,22,23,24,25,26,27,28,29,30,31,32,33,34,35,36,37,38]. At the earliest, two to four weeks after the first pathogen contact, antibodies of the IgM class can be detected in humans; after six to eight weeks, the detection of IgG antibodies is also possible [39,40,41]. *B. burgdorferi*-specific IgM antibodies reach their maximum concentration at about six to eight weeks and then gradually decrease again. In some cases, however, they are detectable for a longer time, which, in turn, makes it difficult to conclude the time of infection. In humans with persistent infection, *B. burgdorferi*-specific IgG antibodies increase slowly over months to years until a plateau value is reached and then remain relatively constant for a long time [42]. There are only a few tick species of the genus *Ixodes* that can transmit the different species of *Borrelia* causing LB: *Ixodes ricinus* is the main vector for LB in Europe [43], whereas in the USA, it is mostly *I. scapularis* and *I. pacificus* [44,45] and in Asia/Eurasia *I. persulcatus* [46].

Both direct and indirect pathogen detection methods are available; however, the direct detection of *Borrelia* spp. by culture is difficult and expensive [47]. The detection of *Borrelia*-specific DNA from the skin, synovial membranes, and other tissue samples is possible using polymerase chain reaction (PCR) during most stages of the disease [48,49]. However, various in-house PCR tests have not been clinically and diagnostically validated under standardized study conditions or standardized as methods for detecting *Borrelia* spp. from various sample materials. Furthermore, the extraction and amplification methods and the selected target genes vary significantly between different laboratories and protocols [47]. Therefore, the value of a PCR for the detection of *Borrelia* spp. in routine diagnostics remains limited [50]. Serological methods for detecting specific antibodies against the representatives of the *B. burgdorferi* complex remain a sensitive, inexpensive, and fast laboratory diagnostic method in both human and veterinary medicine. However, a specific positive antibody level without corresponding clinical signs is not a sufficient criterion for diagnosing LB [47]. Enzyme-linked immunosorbent assays (ELISAs), immunofluorescence antibody tests, Western blots, and the equine multiplex assay as a one- or two-tiered test are most commonly used in equine medicine [5]. The interpretation of positive serological results should always be based on clinical findings and medical history. Other possible causes for the clinical changes should be ruled out [5]. Serological testing in two steps is recommended for the serological diagnosis of equine LB. Firstly, a sensitive screening test (e.g., ELISA) should be used and, as a second test, a highly specific immunoblot in which the sera that were classified as positive in the ELISA are characterized in more detail [51]. The gold standard method for LB diagnosis is a two-step procedure with a sensitive screening test (ELISA) and a specific confirmatory test (immunoblot). However, a study in humans who were treated with antibiotics in the early disease process and developed chronic LB concluded that the presence of chronic Lyme disease could not be excluded by the absence of antibodies against *B. burgdorferi*. Furthermore, they showed that a specific T-cell blastogenic response to B. burgdorferi is evidence of infection in seronegative patients with clinical indications of chronic Lyme disease [52].

Equine granulocytic anaplasmosis (EGA) caused by *Anaplasma phagocytophilum* was first described in the USA in 1969 [53] and has subsequently been reported in Europe (including Germany), Israel, and Brazil [54,55,56]. Whereas EGA is endemic in some regions, it does not occur in others [57]. Seroprevalence studies in horses have been performed in various European countries (Denmark, France, Italy, Sweden, Spain, and Czech Republic), with seroprevalence rates ranging from 7% (Spain) to 73% (Czech Republic) [13,28,33,58,59,60,61,62,63].

The in vitro cultivation of *A. phagocytophilum* from equine blood is rarely pursued because it is difficult, time-consuming, expensive, has a low diagnostic utility [64], and does not provide reliable results at every stage of the disease [64,65]. The detection of typical *A. phagocytophilum* inclusion bodies (morulae) in the cytoplasm of neutrophilic and sometimes eosinophilic granulocytes provides a solid basis for the diagnosis of EGA [53,66,67]. The detection of *A. phagocytophilum*-specific DNA via PCR is a sensitive and specific method [68,69,70], especially in the early and final stages of the disease, since the microscopic detection of morulae at these stages is often not possible [71,72]. Indirect pathogen detection methods for diagnosing EGA, such as ELISA and immunofluorescence tests, are also available [25,62]. A four-fold increase in the titers of specific antibodies over four weeks allows a reliable diagnosis [57,73]. Specific antibodies against *A. phagocytophilum* can be detected from day 14 to 730 post-infection [71,74].

Only limited data on the seroprevalence of *B. burgdorferi* and *A. phagocytophilum* in horses is available in Germany. Similar to *B. burgdorferi*, the seroprevalence of *A. phagocytophilum* varies according to the distribution of infectious ticks and the habitat of the horses (Table 1). Studies performed in Bulgaria reported antibodies against both *B. burgdorferi* and *A. phagocytophilum* in 6.3% (*n* = 192) and 7.1% (*n* = 155), respectively [75,76].

Therefore, this cross-sectional study aimed at determining the seroprevalence of *B. burgdorferi* and *A. phagocytophilum* in the German horse population generally, possible regional variations, and differences in the seroprevalence in horses with a clinical suspicion of LB compared to healthy horses of the same age and from the same stable.

## 2. Material and Methods

### 2.1. Study Group

This cross-sectional study was a co-operation between the equine clinic of the Freie Universitaet Berlin and the Institute for Infectious Medicine and Zoonoses of the Ludwig-Maximilians-Universität München (LMU). The blood samples of horses showing clinical changes typical of LB and clinically healthy animals that resided in the same stable were collected and analyzed. The study was approved by the Landesamt für Gesundheit und Soziales (LAGeSo) in Berlin, Germany (A 0284/17).

The inclusion criteria for clinically suspected animals comprised a suspected diagnosis of equine LB by a veterinarian and the possibility of tick exposure confirmed by the horse owner. In addition to residing at the same stable as the suspected animals, the inclusion criteria for the clinically healthy horses involved the absence of any clinical issues during the previous six months, according to the veterinarian. The responsible veterinarian and the horse owner were asked to participate in a survey, which was available online (LimeSurvey GmbH; Version 2.56.1 + 161118, Hamburg, Germany). It included questions about the clinical signs and the general health status, previous diagnostics, the therapy initiated, and its success.

### 2.2. Serological and Hematological Analysis

Approximately 5 mL of EDTA blood and 10 mL of serum were collected from each horse. All blood samples were analyzed hematologically (inclusion of bodies in EGA in the blood smear) and serologically (detection of *Borrelia* spp. and *Anaplasma* antibodies). A quantitative kinetic ELISA, a SNAP^®^ 4Dx test (IDEXX Laboratories Inc., Westbrook, ME, USA), and a line immunoblot test system (LIA; *Borrelia* Veterinary plus OspA LINE, Sekisui Virotech GmbH, Rüsselsheim, Germany) based on an IgG immunoblot were used for the serological examination. The *Borrelia* spp. antigens DbpA Mix, Osp A Mix, OspC Mix, p39 (BmpA), p58, p83, and VlsE Mix horse were detected in the LIA.

### 2.3. Statistical Analysis

Statistical analysis was performed with the IBM SPSS Statistics program (SPSS Inc., Chicago, IL, USA, version 25). Multiple answers were possible for most questions in the questionnaire. Regarding questions with multiple possible responses, multiple answer sets were defined using the method of multiple dichotomies. A significance level of *p* ≤ 0.05 was set.

Skewness, kurtosis, and the standard error of the mean, median, and mean, as well as graphics (histograms and QQ plots) and statistical test methods, such as the Shapiro–Wilk and Kolmogorov–Smirnov test, were used to check the normal distribution of the data. In addition, frequency tables, bar charts, and pie charts were considered, and information on the measures of location scales was also used for the descriptive analysis.

The chi-square test was used for the association analysis, and Fisher’s exact test was used if the prerequisites for the chi-square test were not met. Furthermore, the odds ratio was determined. Finally, the age distribution was examined using a one-factorial ANOVA regarding the serological *B. burgdorferi* antibody findings.

## 3. Results

### 3.1. Study Group

The blood samples of 236 horses aged 6 months to 30 years were collected from May 2017 to August 2018. The samples were collected by veterinarians throughout Germany, except for the samples of four patients presented directly to the equine clinic of the Freie Universitaet Berlin.

The study population included 123 suspect (symptomatic) and 113 clinically healthy animals. The healthy horses could be used as clinically healthy animals more than the suspect horses from the same stable. Serological examinations were performed on all blood samples collected. All inclusion criteria were fulfilled in 123 suspect horses (blood samples and completed veterinary and owner survey) and 113 clinically healthy horses. These data were included in the statistical evaluation of the questionnaires.

Not all data were available for all horses.

The clinically suspect animals (*n* = 113) were 12.5 ± 5.7 years old, and the clinically healthy horses (*n* = 101) were 13.6 ± 5.0 years old. The years of life were normally distributed in both groups.

**Figure 1:**
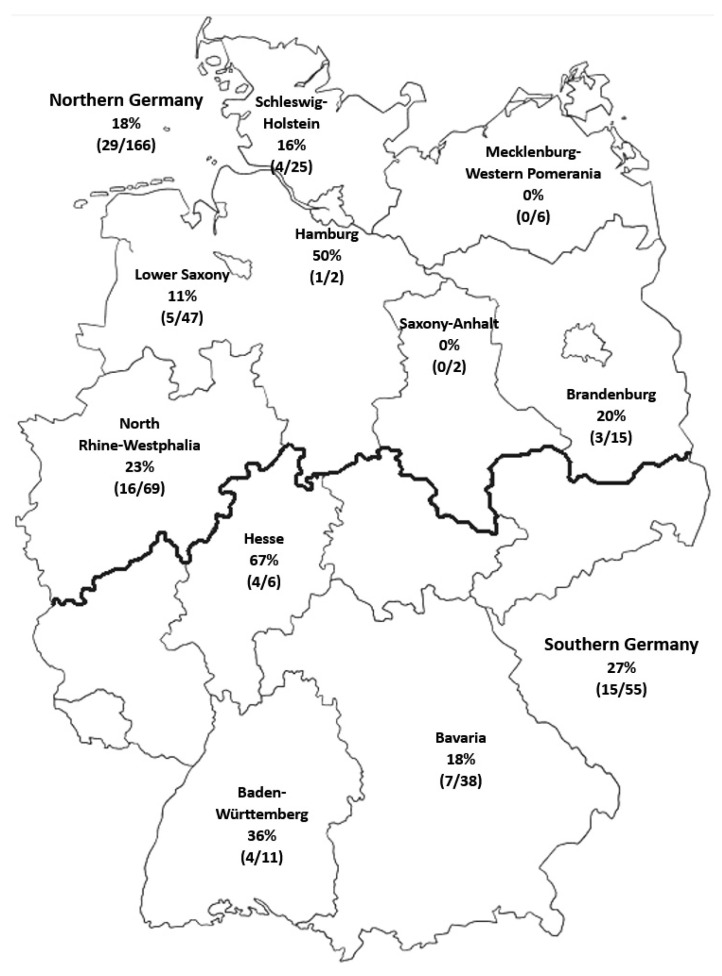
Percentage (%) of *B. burgdorferi* seropositive results with number of horses tested positive and total number of horses tested in the federal state (*n*/*n*; *n* = 221).

Most of the horses resided in northern Germany (86/114; 75.4%; North Rhine-Westphalia, Lower Saxony, Schleswig—Holstein, Brandenburg, Mecklenburg—Western Pomerania, Hamburg, Saxony—Anhalt); the rest were from southern Germany (28/114; 24.6%; Bavaria, Baden-Wuerttemberg, and Hessia), with central Germany remaining under-represented (Figure 1). Slightly more than half of the horses were kept in stable housing (52.6%), followed by those held in open stables (26.3%), exclusively on pasture (15.8%), and in an exercise pen (3.5%).

### 3.2. Results of the Serological Examination

SNAP^®^ 4Dx test (Sensitivity: *A. phagocytophilum* 94.1% and *B. burgdorferi* 95.5%; Specificity: *A. phagocytophilum* 98.4% and *B. burgdorferi* 99.4%): this rapid test was used for the semi-quantitative detection of the specific immune reactions to *A. phagocytophilum* and *B. burgdorferi*. The *A. phagocytophilum*-specific antibodies were detected in 19.5% of the suspect and 16.8% of the clinically healthy horses, and C6 (*B. burgdorferi*)-specific antibodies were detected in 46.3% of the clinically suspect and 46.9% of the clinically healthy animals.

Kinetic ELISA (KELA): the *B. burgdorferi* IgG kinetic ELISA detected KELA units for clinically suspect and clinically healthy horses. These KELA units result from an automatic optical density conversion from the absorbance measurement used in the kinetic ELISA. The mean value of the KELA unit was 259.2 ± 154.2 for the clinically suspect and 254.8 ± 136.9 for the clinically healthy animals. KELA offers an indirect possibility to quantify existing antibodies in comparison to an antibody titer determination. No cut-off value has been defined for horses yet.

Therefore, an average KELA score of 250 was used to subdivide the groups to classify the KELA test results. A statistically significant difference between the evaluation groups “seropositive,” “borderline”, and “seronegative” was determined (*p* < 0.0001). Additionally, higher KELA values (>250) were found in the *B. burgdorferi* seropositive group regarding the results of the LIA (40% had KELA values > 250) than in the borderline group (30.9% KELA > 250) and the seronegative group (29.1%; KELA > 250).

Line Immunoassay (LIA): the results of the “*Borrelia* Veterinary plus OspA Line Immunoblot” used in the study are displayed in Table 2.

Furthermore, 35 of the 123 clinically suspect and 32 of the 113 clinically healthy horses were tested for OspA antibodies (indicative of vaccination status) with the LIA. There were two “+++” ratings for the suspect horses (5.7%) and two “+” ratings for the clinically healthy animals (6.3%). All of these horses were vaccinated against *B. burgdorferi*.

An overall assessment of the serological *B. burgdorferi* result was made based on the LIA. Half (118; 50%, *n* = 236) of the total blood samples examined (total population = suspect + clinically healthy horses) were evaluated with the finding “no suspicion of pathogen contact” (negative), 74 (31.4%) with the finding “indicative of pathogen contact” (borderline), and 44 (18.6%) with the finding “evidence of infection” (positive). Looking at the two different groups, the following resulted: a total of 51% (*n* = 63) of the clinically suspect and 49% (*n* = 55) of the clinically healthy horses tested negative, 28% (*n* = 34) of the clinically suspect and 35% (*n* = 40) of the clinically healthy horses tested borderline, and 21% (*n* = 26) of the clinically suspect and 16% (*n* = 18) of the clinically healthy horses tested positive for *B. burgdorferi* antibodies (*p* = 0.371).

*Anaplasma phagocytophilum*: the number of *A. phagocytophilum*-seropositive horses (clinically suspect 20.2%; *n* = 32; clinically healthy 15.9%; *n* = 17) and horses with evidence of co-infection with both pathogens (suspect 6.1%; *n* = 7; clinically healthy 1.9%; *n* = 2) was higher in the clinically suspect than the clinically healthy population, although this was not statistically significant (*p* = 0.108, Table 3).

Topographical distribution: based on the information on the stables’ location, the seroprevalence distribution in the different federal states was assessed (Table 4). The *B. burgdorferi* seroprevalence of the clinically suspect group (*n* = 114) from southern Germany was 39% (*n* = 11) and 15% (*n* = 4 animals positive for *B. burgdorferi*-specific antibodies) in the clinically healthy group (*n* = 107). The *B. burgdorferi* seroprevalence in the group of clinically suspect horses in northern Germany was 17% (*n* = 15) and 18% (*n* = 14) in the group of clinically healthy horses (Table 4).

Summarizing the findings of all horses, including information about their location provided (*n* = 221), resulted in a *B. burgdorferi* seroprevalence of 27% (15/55) for southern Germany and 18% (29/166) for northern Germany. The federal states with the highest *B. burgdorferi* seroprevalence were Hesse (67%), Hamburg (50%), and Baden-Württemberg (36%). The federal states with the lowest *B. burgdorferi* seroprevalence were Mecklenburg-Western Pomerania (0%), Saxony-Anhalt (0%), and Lower Saxony (11%).

### 3.3. Statistical Analysis

No significant differences between the groups were found when comparing the serological findings of the suspect and clinically healthy horses. However, there was a statistically significant difference between serostatus and age (*p* = 0.011): the median age of seronegative horses was 13 years (1–25 years) and 15 years (5–23 years) for seropositive animals. Horses with borderline serological results had a median age of 11 years (6–30 years).

Forty-two of the participating horses that were suspected of having LB had the same serostatus as their paired clinically healthy animals. The constellation suspect horse positive and clinically healthy horse negative or clinically suspect horse negative and clinically healthy horse positive occurred in 17.8% (*n* = 19) of the cases. The rest of the pairs (*n* = 46; 43.0%) had a *B. burgdorferi* serostatus that differed by one gradation, which means that, for example, the suspect horse had a positive and the clinically healthy horse a borderline *B. burgdorferi* serostatus.

Statistically significant group differences were found between horses with and without cranial nerve deficits (*p* = 0.030), clinical signs of meningoencephalitis and no clinical signs of meningoencephalitis (*p* = 0.003), and simultaneous serological evidence of co-infection. These clinical changes were found more frequently than expected in the co-infected animals.

## 4. Discussion

The present study provides valuable data on the seroprevalence indicative of *B. burgdorferi* and *A. phagocytophilum* infection in German horses and its geographical distribution. Strictly speaking, the serological findings collected are not seroprevalences without bias. The preselection of the sampled population was performed because the subjects of the suspect group showed a suspicion of LB, and also, the group of clinically healthy animals was not randomly selected. Not only was it necessary for at least one horse in the same barn to show clinical signs of LB to participate in the study, but the prerequisite for eligibility as a clinically healthy animal was also to be judged as “clinically healthy”. However, the bias was reduced because clinically healthy horses (most probably representing a cross-section of the German horse population) were included in the study. In addition, the serological findings of the clinically healthy group may assist in the interpretation of positive “LB tests” in clinically healthy horses. The sole evidence of *B. burgdorferi*-specific antibodies has only limited significance if only nonspecific clinical signs are present [47] and must therefore be interpreted with caution [82].

For an approximation of the actual seroprevalence, the clinically healthy group is best suited, as it represents a cross-section of the horse population. In addition, the serological findings of this group allow an assessment of how the positive LB test results in horses without clinical signs represent clinically healthy horses. Unfortunately, only breed, age, and sex were recorded for the clinically healthy group. Risk factors, such as husbandry, use, and tick prophylaxis, cannot be assessed for this group based on the data collected, which somewhat limits the study results with respect to risk factor analysis.

The use of the KELA and SNAP^®^ 4Dx as highly sensitive tools in *B. burgdorferi* indirect diagnostics combined with the highly specific LIA corresponds to the current gold standard in the diagnostics of equine LB as a two-tier test [83] because the important detection of antigens, such as VlsE and C6, are included [84]. In addition, reliable results can be generated for *A. phagocytophilum*-specific antibody diagnostics [85]. The horses classified as seropositive showed high KELA values (>250), but the seronegative animals had low values (<250). This result was statistically highly significant (*p* = 0.000).

SNAP^®^ 4Dx can be used as a screening test but should be checked by immunoblot and classified in more detail if positive [5,77].

In addition to specific antibodies against the *B. burgdorferi* C6 antigen, SNAP^®^ 4Dx also detects specific antibodies against *A. phagocytophilum*. Therefore, simultaneous infections with both pathogens may occur independently since the antibodies against *A. phagocytophilum* and *B. burgdorferi* remain detectable for more extended periods [86,87,88,89], or persistent infection may occur [90,91].

Diagnostic procedures may vary significantly between laboratories [30,31]. It is difficult to give an indication of the sensitivity and specificity of the test methods used since, in *B. burgdorferi* diagnostics, there is not one gold standard in diagnostics with which to compare the results [5].

For *A. phagocytophilum* diagnostics, IFAT for the detection of antibodies against *A. phagocytophilum* can be used as a reference. The relative sensitivity and specificity of the commercially available ELISA (SNAP^®^ 4Dx, IDEXX Laboratories Inc., Westbrook, Maine, USA) when compared with IFAT for the detection of antibodies against *A. phagocytophilum* are 87–100% and 100%, respectively [36,84]. No sensitivity and specificity can be given for KELA, as no clear classification of the results into seropositive, limited, or negative was made here.

Determining highly specific antibodies in a species-specific immunoblot is of great importance in *B. burgdorferi* diagnostics [92]. In this study, horses were classified as *B. burgdorferi*-seropositive if the immune reactions evaluated suggested the presence of an active *B. burgdorferi* infection. Accordingly, 26 horses (21%) from the clinically suspect and 18 horses (17%) from the clinically healthy group had a positive result. Just over half (51%) of the suspect and just under half (49%) of the clinically healthy horses tested negative, whereas 28% of the suspect and 35% of the clinically healthy horses tested borderline for *B. burgdorferi* antibodies. Therefore, the serostatus of the clinically healthy horses hardly differed from that of suspect horses (*p* = 0.887).

Consequently, it can be concluded that the detection of specific antibodies against *B. burgdorferi* alone is not sufficient for a diagnosis of equine LB, which is consistent with the results of Divers et al. [5]. Evidence of previous infections with pathogens of the *B. burgdorferi* complex was found even in a relatively high proportion of clinically healthy horses. This finding reflects the observation of other authors that horses regularly deal with the pathogen without the development of clinical signs [93,94,95] and with the observations from human medicine that high antibody levels against *B. burgdorferi* can be found particularly in people with high pathogen exposure [96]. Similar to horses, a diagnosis of LB in humans can only be made when the typical clinical signs are observed. A corresponding causal therapy alleviates the manifestation of clinical signs [96,97]. Highly specific, highly sensitive qualitative, and semi-quantitative direct detection methods or the analysis of the T-cell response against *B. burgdorferi*, as described by Dattwyler in humans [52], would help remedy this situation but do not appear to be near at hand.

In this study, *A. phagocytophilum*-specific antibodies were detected in 19.5% of the clinically suspect and 16.8% of the clinically healthy horses. Furthermore, co-infection with *B. burgdorferi* and *A. phagocytophilum* was found in seven suspect and two clinically healthy animals. Egenvall et al. [28] were able to show a possible association between the seropositivity of both pathogens and host factors, such as gender, age, breed, use and exposure to ticks, and clinical signs. The two titers were correlated significantly, and it can be assumed that a previous infection or co-infection with *A. phagocytophilum* may lead to immunosuppression and, thus, might promote *B. burgdorferi* infection [98,99]. On the one hand, other studies have not been able to link such a connection between the specified host factors and seroprevalence [100]. Then again, an age dependency was also found in the present study: age and seroprevalence correlated positively, which agrees with the results of other authors [28]. Since *B. burgdorferi* antibodies persist over extended time periods [86], a higher average age with a positive finding can be explained because older horses have a higher probability of previous contact with the pathogen.

The *B. burgdorferi* seroprevalences observed are challenging to compare depending on the country of origin, the number of samples, and the varying determination methods. The immunofluorescence antibody test is commonly used, but it often delivers false-positive results due to its low specificity [101]. It is now considered an unsuitable diagnostic tool due to the lack of comparability of the results [39,102] and the inability to distinguish between vaccinated individuals and field infections [103]. The unspecific serological testing of horses in endemic areas is critical as it leads to an overdiagnosis of equine LD [82].

Another similar study performed in Germany determined an overall seroprevalence of 13.1% in Germany [79], which is similar to the seroprevalence in this study. The intention of our study was to screen horses throughout Germany for infection with the tick-borne diseases Lyme borreliosis and equine granulocytic anaplasmosis. A wide range of data not limited to the geographic origin and symptoms of the subjects was collected and included tick exposure, prophylactic measures, health status, and previous diagnostics and therapy. Our results clearly showed that horses in Germany may have been infected with *B. burgdorferi* but do not necessarily develop clinical signs.

## 5. Conclusions

Since the relevance of a positive antibody titer against these pathogens remains to be elucidated, the unspecific testing for antibodies against *B. burgdorferi* without a definite clinical suspicion of LB is not recommended.

## Figures and Tables

**Table 1:** Seroprevalence of *Borrelia burgdorferi* and *Anaplasma phagocytophilum* in European countries.

Country	*B. burgdorferi* Seroprevalence	*A. phagocytophilum* Seroprevalence
Austria	15–93% [30]	
Belgium	22% [77]	
	22% [38]	
Czech Republic		72.8% [63]
Denmark	29% [33]	22.3% [33]
France	23.7–52% [78]	11.3% [58]
	13.5% [59]	
Germany	14.5% [79]	
	0–64% [80] (focus on Bavaria)	
	3.3–61.1% [31]	
	47.9% [10]	
	27–48% [81]	
	0–16.1% [21]	
Italy	2.3–7% [13]	13.4% [13]
	5.1–15.3% [36]	17% [60]
	24.3% [35]	
Spain		6.5% [62]
Sweden	16.8% [28]	16.7% [28]
The Netherlands		9.8% [61]

**Table 2:** Results of the line immunoassay in 123 suspicious and 113 clinically healthy horses.

Antigen	Suspect Horses (*n* = 123)		Clinically Healthy Horses (*n* = 113)	
	Negative	Positive	Negative	Positive
DbpA Mix	102	21	95	18
OspC Mix	97	26	99	14
p39	87	36	81	32
p58	98	25	33	80
p83	39	84	33	80
VlsE Mix	64	59	58	55

**Table 3:** Serological results compared between *Borrelia burgdorferi* and *Anaplasma phagocytophilum*.

	*Borrelia burgdorferi*			*Anaplasma phagocytophilum*		Co-Infection	
	Negative	Borderline	Positive	Negative	Positive	Negative	Positive
Suspect horses (*n* = 114)	56	32	26	91	23	107	7
Clinically healthy horses (*n* = 107)	51	38	18	90	17	105	2

**Table 4:** Distribution of the *Borrelia burgdorferi* sensu lato seroprevalence in the federal states of Germany.

Region	Federal State	Clinically Suspect (*n* = 114)			Clinically Healthy (*n* = 107)		
		Negative	Borderline	Positive	Negative	Borderline	Positive
Southern Germany	BW	2	1	3	4	0	1
	BY	8	5	6	8	10	1
	HE	1	0	2	1	0	2
	Total	11	6	11	13	10	4
Northern Germany	BB	4	3	1	2	3	2
	HH	0	0	1	1	0	0
	MV	1	2	0	2	1	0
	NI	17	3	4	10	12	1
	NRW	14	15	7	18	6	9
	SH	8	3	2	5	5	2
	ST	1	0	0	0	1	0
	Total	45	26	15	38	28	14

BW: Baden-Württemberg, BY: Bavaria, HE: Hesse, BB: Brandenburg, HH: Hamburg, MV: Mecklenburg-Western Pomerania, NI: Lower Saxony, NRW: North Rhine-Westphalia, SH: Schleswig-Holstein, ST: Saxony-Anhalt.

## Data Availability

Further Data can be obtained from the corresponding author.
